# Reminiscence Therapy and Music With Older Adults: A Descriptive Study Investigating the Current Views and Practices of Australian Aged Care Providers and Volunteers

**DOI:** 10.1177/07334648241236236

**Published:** 2024-02-29

**Authors:** Romy Engelbrecht, Sunil Bhar, Helen Shoemark, Bradley Elphinstone, Joseph Ciorciari

**Affiliations:** 1 School of Health Sciences, Department of Psychological Science, 3783 Swinburne University of Technology, Melbourne, VIC, Australia; 2 Department of Music Therapy, Boyer College of Music and Dance, 6558 Temple University, Philadelphia, PA, USA; 3 Centre for Mental Health, 3783 Swinburne University of Technology, Melbourne, VIC, Australia

**Keywords:** caregiving, memory, mixed methods, music, psychology

## Abstract

Reminiscence therapy and music are often used to improve the wellbeing of older people; however, we do not know how these interventions are used in practice. This study explored how those working with older people view and use verbal Reminiscence Therapy (VRT) and Music-assisted Reminiscence Therapy (MRT). A total of 110 participants who worked or volunteered with older people in Australia were surveyed in this descriptive, mixed-method study. VRT and MRT were frequently and spontaneously used to respond to the varied needs of older adults. VRT and MRT lead to reported positive outcomes including better care practices, positive affect and mood, and improved social connections. MRT was used as a compensatory strategy when traditional VRT was not possible. This study describes the current practices of VRT and MRT, and an overview of how reminiscence-based approaches are used in Australia to address the health and wellbeing of older people.

**What this paper adds**

• The study describes the *treatment as usual* practices for reminiscence therapy and music-assisted reminiscence therapy by care workers in Australia.
• Details the frequency of use, implementation methods, outcomes, and topics of discussion for both interventions.
• Both interventions were widely used by all occupational groups and viewed as successful by staff.

**Applications of study findings**

• Informs clinical practice by encouraging the use of music in reminiscence therapy approaches.
• Validates the use and implementation methods of both interventions for future research across occupational groups.
• Provides indicators for the clinical situations in which MRT may be useful as an intervention over and above VRT (e.g., as a compensatory strategy).

## Introduction

The psychological wellbeing of older adults directly impacts their physical health, functional and cognitive decline, and life expectancy (Steptoe et al., 2015). It is imperative that all aged care providers have effective strategies to support the mental health of the aging population. Reminiscence therapy and music-assisted reminiscence therapy could represent effective and useful mental health interventions for those caring for older adults.

Reminiscence therapy (RT) is an intervention that uses purposeful and directed reminiscence to improve wellbeing (Webster et al., 2010). RT has been found to be effective in improving mood, cognitive function, loneliness, quality of life, mental health, happiness, and wellbeing for older people in care and living at home (Bohlmeijer et al., 2007; Brooker & Duce, 2000; Chaing et al., 2010; Gudex et al., 2010). Different types of RT exist, each with different purposes and outcomes on the wellbeing and psychological adjustment of older people (Wong & Watt, 1991). When using the model developed by Webster and colleagues (Webster et al., 2010) RT can take the form of simple discussions around a specific topic, such as “holidays” (*simple* reminiscence), a review of one’s life focused on finding meaning and identity (*life review*), or a reflection of past problem solving successes and achievements (*life review therapy*).

Music-assisted RT (MRT) is the use of music in any form (e.g., recorded, live, or as a topic for discussions) to enhance the reminiscence therapy experience (Engelbrecht et al., 2021). MRT makes use of several benefits associated with therapeutic music, including helping to summon autobiographical memories, express and experience emotions, elicit physiological responses, and define and express self-identity and social relationships (Engelbrecht et al., 2021). The inclusion of therapeutic music in RT has been found to improve psychological wellbeing of older adults above and beyond that of RT alone (Istvandity, 2017). MRT has resulted in improvements to mood, depression, anxiety (Ashida, 2000; Mahendran et al., 2017; Mohammadi et al., 2011; Rawtaer et al., 2015), group coherence, life satisfaction (Haslam et al., 2014), increased memory recall (El Haj et al., 2011); strength of emotional responses; and speed, vividness and pleasantness of recalled memories (El Haj et al., 2012). Furthermore, a recent pilot study demonstrated that MRT was a feasible, acceptable and potentially useful strategy to improve psychological distress for older adults (Engelbrecht et al., 2022).

It is unclear how reminiscence therapies with and without music are being used in practice, or by whom. The use and implementation methods of verbally delivered RT (RT delivered without music; VRT) and MRT in research is varied and often poorly reported (Istvandity, 2017), with these interventions delivered in groups and individually over single session designs (James & Bhar, 2016), time-limited programs from 6 up to 12 weeks (Ashida, 2000; Cuevas et al., 2020; Duffey et al., 2008; Haslam et al., 2014; Mohammadi et al., 2011; Stinson, 2009), or as part of the ongoing lifestyle program (Mohammadi et al., 2011; Rawtaer et al., 2015), across different populations. Only one study to date has provided insight into the usual practices of such interventions. Kris et al. (2017) surveyed 43 caregiving nurses working at nursing homes in Connecticut, USA, and found that less than half frequently engaged in reminiscence with their clients.

Given the paucity of such research, the aim of this study was to quantify and qualify the usual practices of all aged care providers with respect to using VRT and MRT. Such an exercise was expected to clarify the “*treatment as usual*” paradigms for those in aged care in Australia. The aims of this study were to investigate: (1) The extent to which VRT and MRT were used by those interacting with older adults and providing care in the aged care sector, (2) How such interventions were delivered and viewed, and (3) The purpose and observed benefits of such interventions.

## Method

This study involved a mixed-method design, involving quantitative data collection integrated with qualitative data analysis. This study was descriptive; therefore, there were no hypotheses.

### Participants

Individuals were eligible to participate if they were 18 years or over, working in a paid or volunteer capacity with adults aged 65 years and over, and were able to read and speak English. The sample comprised 110 participants. Of these, 18 participants did not answer any questions after consenting to participate, and were removed for analysis. The sample analyzed comprised the remaining 92 participants (See Table 1 for participant demographic information).

**Table 1.** Participant Demographic Information.

	Participant variables	Mean (SD)	N (%)	Range
Age (years)		42.6 (14.4)		19–79
Gender	Male		17 (19%)	
	Female		74 (81%)	
Language spoken at home	English		86 (95.6%)	
	Other		4 (4.4%)	
Years of work experience		8.5 (7.63)		0.5–40
Occupation	Personal carers		8 (8.7%)	
	Nurses		22 (22.8%)	
	Doctors		1 (1.1%)	
	Allied health total		41 (44.6%)	
	Art therapists		4 (4.3%)	
	Audiologists		1 (1.1%)	
	Dentists		1 (1.1%)	
	Music therapist		11 (12%)	
	Neuropsychologists		2 (2.2%)	
	Occupational therapists		3 (3.2%)	
	Pastoral carers		3 (3.2%)	
	Physiotherapist aid		1 (1.1%)	
	Psychologists		12 (13%)	
	Social workers		3 (3.2%)	
	Volunteers		5 (5.4%)	
	Lifestyle/diversional therapists		6 (6.5%)	
	Other		10 (10.9%)	
Work setting*	RACF		48	
	Community		17	
	Hospitals		35	
	Palliative care		27	
	Private clinics		8	
Location in Australia	VIC		54 (59.4%)	
	NSW		15 (16.5%)	
	QLD		6 (6.6%)	
	SA		2 (2.2%)	
	WA		0	
	NT		0	
	TAS		3 (3.3%)	
	ACT		11 (12.1%)	

*Note.* participants could select multiple work settings.

All workers and volunteers were included in the sample and analyzed to reflect genuine use of these interventions across interdisciplinary practice. The allied health sample comprised workers such as psychologists, social workers, pastoral carers, music therapists, occupational therapists, and art therapists. Lifestyle workers, otherwise known as diversional therapists, are personal carers who specialize in designing and delivering leisure and social and therapeutic activities to individuals and groups in aged care. Participants could select multiple work settings, and worked across settings such as residential aged care facilities (RACF), community care, or hospitals. The majority of participants (66%) worked in one setting only (e.g., residential aged care only).

### Materials

Data were collected using a purpose-built survey. Items were initially created based on existing theory and literature, and refined through two small focus groups with a total of six allied health workers in aged care (from psychology, music therapy, social work, and pastoral care) who consented to participate and completed the survey in the presence of the researcher. The aim of these groups was to help improve the clarity, suitability, and accuracy of the survey.

The final survey comprised 20 multiple choice and 8 open-ended questions. The survey took approximately 20 minutes to complete. Multiple-choice questions were used to collect demographic information, and assess if participants delivered VRT and MRT, the frequency of such delivery, the mode of delivery (group, individual, or both), and if prompts were employed to trigger memories (e.g., objects or pictures). Open-ended questions on VRT and MRT asked participants to define the interventions, provide reasons for their use, identify barriers to implementation, and describe a situation when they have used them with an older person.

### Procedure

The survey (online or hardcopy) was distributed to two large national aged care provider organizations, three special interest professional social networking groups, two education providers and professional bodies through an email, online post, flyer, or in-person. Multiple distribution methods were used to maximize the number of potential participants. Data were collected between October 2018 and August 2019. As an incentive, participants were offered the opportunity to win a $250 gift card in a lottery draw. This study was approved by Swinburne’s Human Research Ethics Committee (2018/258).

## Planned Analysis

Data analysis strategies for this study were exploratory. Comparisons were made between the variables of frequency and type between VRT and MRT methods using descriptive statistics. Occupational groups were used for the analysis of frequency of use and type of RT, including all, nurses, carers (personal care assistants and lifestyle carers), allied health, and volunteers and others. Preliminary logistic regression analysis showed that no other demographic variables predicted the use of VRT or MRT. Quantitative data were analyzed using IBM SPSS (version 29).

The qualitative data generated by the open-ended questions were analyzed using an inductive content analysis approach (Saldaña, 2009), allowing the themes and codes to be identified from the data. Coding of participant responses was conducted using Quirkos software with manual “in vivo” coding of the participants’ language and terms used, and pattern coding to group similar terms and language together for situations of use, topics of discussion, and outcomes were used to code responses (Saldaña, 2009). Rigor and validity were ensured by collecting data via written open text responses, thereby avoiding interviewer effects or biases. Open questions were answered prior to the multiple-choice questions regarding RT and MRT to ensure participant responses were not primed. Qualitative data were triangulated with an external researcher experienced in qualitative research, who independently coded the data, and met with the leading author to reach consensus with the coding and ensure rigor of the findings.

## Results

### Quantitative Data

Overall, a total of 78 (84.7%) respondents reported using VRT in their work, with sixty-two participants reportedly using MRT (reminiscence involving the discussion, listening, or making of music) in their work, 82% of those who reported using VRT. A minority (*n* = 13, 17.3%) of participants reported “*never*” or using music in RT (see Table 2).

**Table 2.** Reported Frequency of VRT and MRT Use for Staff According to Occupation.

	All		Nurses		PCA and lifestyle		Allied health		Volunteers and others	
	*n*	*%*	*n*	*%*	*n*	*%*	*n*	*%*	*n*	*%*
Reported use	VRT use								
Daily	20	22.5	7	36.8	6	42.9	7	17.1	0	0
Several times per week	34	38.2	6	31.6	7	50	19	46.3	2	14.3
Once a week	15	16.9	2	10.5	1	7.1	4	9.8	8	57.1
Once a month	5	5.6	1	5.3	0	0	3	7.3	1	7.1
Rarely	1	1.1	0	0	0	0	1	2.4	0	0
Never	14	15.7	3	15.8	0	0	7	17.1	3	21.4

	All		Nurses		PCA and lifestyle		Allied health		Volunteers and others	
	*n*	*%*	*n*	*%*	*n*	*%*	*n*	*%*	*n*	*%*
Reported use	MRT use								
All the time	15	20	0	0	3	21.4	12	35.3	0	0
Most times	24	32	6	37.5	6	42.9	8	23.5	4	36.4
Sometimes	17	22.7	4	25	5	35.7	5	14.7	3	27.3
Occasionally	6	8	2	12.5	0	0	4	11.8	0	0
Never	13	17.3	6	25	0	0	5	14.7	4	36.4

Across occupational groups, the majority of staff reported using VRT *“Daily*,” *“several times per week*,” or “*once a week”* (see Table 2). MRT was used by the majority of all occupational groups “*most times”* or “*sometimes”* when using RT (see Table 2). Overall, music (*n* = 62, 53.2%), pictures (*n* = 55, 49.5%), and objects (*n* = 41, 36.9%) were the most used prompts to facilitate RT, followed by technology (*n* = 28, 25.2%) and scents (*n* = 7, 15.3%), while 4.5% of respondents (*n* = 5) reported using no prompts.

For the total sample, the types of music used in MRT included live (*n* = 27, 24.5%), recorded (*n* = 51, 45.9%) and as a topic of discussion (*n* = 39, 35.1%), with respondents often using several types. MRT was most commonly used during reminiscence discussions (*n* = 45.9%) compared to after (*n* = 28.8%), and before (*n* = 23.4%).

Of all participants using VRT, most delivered the treatment in both group and individual contexts (*n* = 43, 57.3%), with less using VRT exclusively in individual (*n* = 28, 32.3%) or group (*n* = 4, 5.3%) sessions. Similarly, of participants using MRT, most delivered the treatment across both group and individual contexts (*n* = 36, 61%), with less using MRT exclusively in individual (*n* = 16, 27.1%) or group (*n* = 7, 11.9%) sessions.

A two-way 2×3 repeated measures ANOVA was used to determine if significant differences in frequency were identified between the approaches of VRT and MRT and types of RT for the different occupational groups. Simple reminiscence was used significantly more in both VRT and MRT than life review, and life review therapy, for most occupational groups, except for nurses, and volunteers and other (see Table 3). MRT was reported to be used significantly less than VRT by nurses, and the total combined sample. There were no significant differences for VRT and MRT use for other occupational groups (see Table 3).

**Table 3.** ANOVA and Post-hoc Comparison Results for the Type and Approach of RT Methods Reported to Have Been Used Over the Last Year by Staff Occupation.

Occupational group	N	Simple RT (1)		Life review (2)		Life review therapy (3)	
		Mean (SD)		Mean (SD)		Mean (SD)	
		VRT	MRT	VRT	MRT	VRT	MRT
All	51	3.76 (1.031)	3.35 (1.110)	2.08 (.744)	1.90 (1.063)	3.02 (.969)	2.45 (1.137)
Nurses	12	3.67 (1.155)	3.00 (0.953)	2.50 (0.798)	1.83 (0.937)	3.17 (1.267)	2.42 (0.900)
PCA and lifestyle	10	4.30 (1.059)	3.40 (1.265)	2.20 (.789)	2.10 (1.101)	3.00 (.816)	2.50 (.850)
Allied health	23	3.52 (.994)	3.43 (1.237)	1.87 (.626)	1.83 (1.154)	3.04 (.928)	2.52 (1.410)
Volunteers and others	6	4.00 (.632)	3.67 (.516)	1.83 (.753)	2.00 (1.095)	2.67 (.816)	2.17 (.983)

ANOVA results	Main effects				Comparisons^b^			Interaction *p*
	VRT versus MRT		Type of RT		1 versus 2	2 versus 3	1 versus 3	
	*F*	*p*	*F*	*p*	*p*	*p*	*p*	
All	10.843 (1, 50)	.002	75.59 (1.792, 89.61)^a^	.000	.000	.000	.000	.142 ns
Nurses	11.252 (1, 11)	.006	11.252 (1, 11)	.006	.002	.042	ns	.968 ns
PCA and lifestyle	4.765 (1, 9)	.057 ns	18.329 (2, 18)	<.001	.003	.026	.012	.122 ns
Allied health	1.162 (1, 22)	.293 ns	32.707 (2,44)	<.001	<.001	<.001	<.001	.310 ns
Volunteers and others	.769 (1, 5)	.421 ns	18.894 (2, 10)	<.001	.014	ns	.030	.206 ns

^a^Sphericity violated, Greenhouse-geisser correction used.

^b^Post-hoc LSD Bonferroni corrected comparisons. 1 = Simple RT; 2 = Life review; 3 = Life review therapy.

### Qualitative Data

The majority of participants described a situation which used VRT and MRT individually, and with older women.

#### Situations of Use

Situations of use was defined as the context in which the intervention was used and described by participants, involving details relating to the format of the intervention, such as type (e.g., type of verbal or musical interaction), purpose, prompts, frequency, timing, or delivery. Four major themes for the situations of use for VRT and MRT were observed.

##### Staff Used VRT and MRT in Response to Immediate Client Needs for Distraction, Emotional or Psychological Processing, or for Social Connection

Participants reported using VRT and MRT with older people who had clear needs (e.g., as a direct treatment of distress). Participants reported distinct patterns of use related to the immediate needs of clients, such as the need for distraction (e.g., articulated as from “pain,” “agitation,” “stress,” “anxiety,” or “confusion”), emotional or psychological processing (during “grief,” “lamenting,” “regrets,” “depression,” and “sadness”), and for social connection (e.g., those who are “shy,” “withdrawn,” “non-verbal,” or experiencing “loneliness”). This was consistent across both VRT and MRT. For example, one participant, a lifestyle assistant reported they:

Used reminiscence with an older person with dementia who was upset. I picked up a photo of her grandsons when they were children from her bedside table and asked her to tell me about them…She recalled humorous stories of them as cheeky little boys and her mood improved.

##### The Use of VRT and MRT was Often Spontaneous, and was Integrated Into Routines of Daily Care

While some respondents described VRT and MRT as planned and intentional interventions, typically participants described situations where VRT and MRT were used spontaneously in addition to other activities or daily routines, such as while assisting older adults with activities of daily living (ADLs). For example, a nurse reported: “Talking to patients when feeding them about their previous occupation, children if any and hobbies.” Participants also used MRT at these times, for example, this nurse:

…Put together a playlist of these songs and during hygiene time (predominantly showering which was a time of high stress and anxiety) we played the music and this allowed for relaxation and positivity. It also “kept the story going” as we added to the playlist over time.

This suggests that VRT and MRT are used as an integrative strategy to support interpersonal and personal care, and can be used in a spontaneous way.

##### MRT Use was Based on Musical Heritage (Musical Preferences, History and Background)

Musical heritage, including individual musical preferences, personal history and musical background was informed by the older person or family, and used to select music for use in RT, for example, a psychologist reported:

I used Spotify on my phone and had selected a number of songs by artists her son informed me she used to like. We listened to songs by The Beatles, The Easybeats and Queen. The music prompted the resident to recall details of concerts she had attended.

##### MRT was Used as a Compensatory Strategy When Clients Were Unable to Verbally Respond

MRT was often used in situations with older people when they were not able to engage in verbal discussions in RT (e.g., those who were “unable to speak,” “non-responsive,” and “non-verbal”), for example, one art therapist described:

An older person with dementia, not able to speak appeared to respond to time I spent with her: assisting with meals, sitting and talking to her or reading to her. When I sit face to face with her and sing she joins me and sings along word-for-word.

MRT was therefore reported as a compensatory and accessible strategy, where music was used as the process of reminiscence.

#### Topics of Discussion

Topics of discussion related to the content, theme, or topic of reminiscence, music or song reported or described by the participants. The topic could be driven by the staff participant, or older person. Three major themes were identified in participants’ descriptions of experiences with MRT and VRT.

##### Relationships

Participants reported that relationships to others were a key concept in the recounted personal narrative by older people, both as a way of acknowledging those of importance, those who have died, and to define ones’ identity by the relationships or role (e.g., as a “*mother*”). For example, the described experience of a psychologist working with a distressed client who refused to leave her room and was constantly waiting for her children to visit. The participant said:

I sat down with her and asked her about her children and her as a mother. What were they like as children? How did she like mothering? I could tell that mothering was a very important part of her life and I reflected that she must have done a wonderful job in her role as a mother to have such successful and loving children…she became very comfortable talking.

##### Places

Participants reported places as a common theme during the reminiscence experience for older adults. Participants reported that older adults reflected on places of origin, culture, or transitions of moving or immigration as connected to identity and to describe ones’ life. One nurse reported:

One lady experiences great general pain, through talking, she shared her long history of working on farms, of experiencing apprehension when the boys in her family went to war, of how the women managed the animals, drought, stock and produce prices, town resources and love of the land, she often asks of the area she lived in, and has family who occasionally visit and share pictures and stories of the town and how much it’s changes and who is still there and other generational news.

##### Identity

Participants reported that older people discussed identity constructs in both VRT and MRT. The use of VRT and MRT involved discussing, acknowledging, or enhancing identity constructs such as personality, personhood, strengths, achievement, and previous roles or work. Identity was also related to the outcomes of VRT and MRT and is therefore discussed further below.

#### Outcomes

Outcomes refer to the participants’ perceptions of results of VRT and MRT. Outcomes could be positive or negative, experienced by staff or clients, and quantified in any way. Participant responses demonstrated four themes related to the outcomes of using VRT and MRT with older adults.

##### Enhanced Social Connections

During VRT use, participants emphasized the shared and collaborative nature of the experience, and the quality of the interaction. VRT also assisted to develop relationships and build further conversation. This enhanced social connection was one of the most prevalent codes in the participant responses. One nurse reflected:

Going through a photo album, talking about who was in the pictures, the fashion, descriptions of where and when the photos were taken. This then assisted with later conversations as it was a reference to return to. The emotions shared, both joy and sadness, was a special time to share.

Further responses indicated the importance of a willingness to be present and to connect, for example, this nurse, “…felt time was needed just to sit there with the patient and get them focusing on something else….” Similar themes were identified in MRT, with the addition of extending to the social benefits with family, for example, this nurses’ experience of MRT lead to enhanced social connections with her children, “It was a journey of wonderful memories and conversation with her children and a part of her life she had not shared with them.”

##### Improved Affect and Mood

Participants reported a range of positive affective changes from VRT interventions, commented on themes of emotional reactions, specific positive affect (e.g., happiness, joy, comfort), and improved mood. For example, a nurse reflected, “The patient enjoyed this as he was able to connect personal moments about his life to someone willing to listen which made him really happy.” This was also a common outcome for MRT. Participant experiences centered around happiness, joy, and relaxation, for example, this lifestyle carer shared:

We use it often we know that the ability to recall and reflect helps older adults remember who they used to be and it also makes them feel good. They get an endorphin flow in the moment. It can calm a stressed resident.

##### Better Care Practices

A variety of benefits were reported in relation to staff behavior. Staff reported reductions in medications administered, agitation, behaviors of concern, and that VRT interventions influenced social programs offered, resulted in improved staff handover and communication, and helped to establish treatment goals and make lasting change. One psychologist described the outcome following VRT the older person was “talking away excited for us to continue to see her. When I spoke to the care manager 2 weeks later, she stated that she had seen a real change.” While another participant (audiologist) used VRT to show “what they remember life being like before their hearing declined in order to establish rehab goals.” Another participant (nurse) noticed the outcome of “…diversional therapy with reminensing [sic] was we didn’t need to give drugs.”

For participants, the inclusion of music facilitated similar and additional changes to care practices, including increased acceptance and success of care, staff learning, changes to social programs, staff handover, learning and communication, and building resources (e.g., playlists or legacy items). For example, a young nurse reflected that in MRT, they were able to use music as common ground to improve care practices as a “conversation topic which helped me to build rapport with this patient. This facilitated my care with them as I believe they were more comfortable to concede health details with me.”

##### Connect to Identity

Participants reported that older people often discussed concepts related to their identity. In discussing these topics, participants perceived that the older people were able to reconnect with their past and present identity or sense of self. This was evident across both VRT and MRT, for example, these two lifestyle carers believed it helped them feel… “connected to their past as this gives them a sense of identity” and “the ability to recall and reflect helps older adults remember who they used to be and it also makes them feel good. They get an endorphin flow in the moment. It can calm a stressed resident.”

##### Negative Outcomes

Two participants, both psychologists, reported negative or unforeseen outcomes of VRT. Firstly, an older person “became mournful.” However, this participant was able to redirect the patient for a good outcome. Another participant commented that when using VRT with older people with dementia that “At times it was difficult to see an outcome due to cognitive impairment, but often it bought a lot of joy to residents.”

Two negative or unforeseen outcomes were described when using MRT. A volunteer participant reported some agitation and unhelpful reminiscence resulting from MRT, as the client “sometimes circles back to his dissatisfaction at being in aged care (non-voluntary—has court appointed guardian). Can also cause some agitation as he then gets frustrated that he can’t play anymore.” Another participant, a care manager, described music eliciting unintended emotions related to a residents’ grief about his wife when they unknowing played his wedding song during MRT. The participant felt that this was appropriate and clinically useful. While not a negative outcome, another participant, a psychologist, reported having trouble and lack of confidence in establishing musical preferences, for example, “A list of songs was put together, but I did not feel confident that the music chosen was exactly right for the resident.”

## Discussion

This descriptive study aimed to clarify how aged care workers used and viewed VRT and MRT strategies, the types of RT used, and the methods of implementation. The findings of this study indicated that VRT and MRT were widely used across occupational groups to improve and promote wellbeing. The majority (84%) of participants used VRT, of which a significant proportion (82%) also employed MRT. It is possible then that MRT is viewed as a regular and additional component of RT by the workforce, rather than a separate intervention entirely. This study provides the first step in understanding the frequency of the use of MRT with older people, with only 17% of the sample participants never using music. Given the benefits associated with the inclusion of music in RT (El Haj et al., 2011, 2012; Haslam et al., 2014; Potts et al., 2020; Rawtaer et al., 2015) music in any form should be embedded or offered within RT in research to reflect treatment as usual.

Despite the high rate and frequency of nurses using MRT, it was used significantly less often. Given numerous studies that demonstrate that music enhances the outcomes of reminiscence, this is surprising. It may be that the identified limitations in staff training, confidence, or access to music are barriers that reduce the uptake of using music in RT for nurses, particularly given the reported spontaneity of its use.

*Simple* RT was used significantly more than *life review,* and *life review therapy* over the last 12 months for both VRT and MRT for most occupational groups. *Life review therapy* was used to a lesser extent. Past research suggested that *life review* and *life review therapy* were the most effective VRT methods for improving wellbeing outcomes for older people, and *simple* RT was the most effective for those with dementia (Bohlmeijer et al., 2007; Pinquart & Forstmeier, 2012). While few overt references to the type of RT were noted within the qualitative data, a distinct pattern of using VRT and MRT emerged related to the particular needs of the clients. A schema was proposed using the participant responses to describe the clients’ needs, including for distraction (from anxiousness, pain, agitation), emotional or psychological processing (during grief, regrets, lamenting), and for social connection (for those experiencing loneliness, withdrawal etc). These can be related to the purposes of *simple* and *life review therapy,* indicating consistency between the qualitative and quantitative data, and that the older persons needs have driven how the interventions were applied. Likewise, overwhelmingly these interventions were reported to be spontaneous and integrated as part of daily care, for example, in addition to feeding or hygiene routines, perhaps explaining why *simple* RT was the most used type.

Both VRT and MRT were perceived as a social experience that was part of everyday care, often accompanying other activities. The care workforce emphasized the quality of the interaction for themselves, with notions of *willingness* to be present and of *sharing over time*, purposefully building on reminiscence storytelling. Clear changes to staff behavior and care practices as a result of using these interventions were also evident within the experiences detailed by the participants. These are perhaps highly valuable and easily implemented interventions that could address the interpersonal needs of the client in aged care settings, that may overcome issues such as the depersonalization of older individuals in institutions (Gudex et al., 2010). This was further supported by the common themes identified within participant responses for both interventions relating to sense of self, people or roles, and places to describe ones’ life and self.

The way in which the interventions were delivered varied. Music was most frequently used *during* RT, compared to *before* or *after*, and in practice, music is flexible in its delivery from live music, recorded music, or as a topic of conversation. Both delivery formats (verbal and music) were also used in both group and individual contexts, rather than in individual only, or group only. These results indicate that VRT and MRT are used clinically across both individual and group contexts in treatment as usual paradigms, and therefore validates both approaches for further research. What remains to be further explored is the type (e.g., live vs recorded) and the size of the dose of music required in RT, and how much VRT and MRT is needed to achieve clinically significant wellbeing outcomes.

One key difference between the interventions was that MRT was used as a compensatory strategy to provide interpersonal care or interaction to those who may not have the capacity to engage in verbal discussions, such as those who are non-verbal or with memory impairments. Music engenders an experience that does not require verbal articulation, and therefore can be the focus in the MRT experiences, and can function at multiple and intrinsic hidden levels that can still shared socially (e.g., as an internal emotional and reminiscence experience) (Engelbrecht et al., 2021).

This study explored the methods and staff views of VRT and MRT in clinical practice. Despite the contribution of the findings, several limitations were evident. The qualitative data was obtained through asking participants to reflect on an experience of using the interventions, and therefore may only reflect one instance rather than typical practice. Data was only collected for self-reported staff behavior; therefore, no conclusions can be drawn on the outcomes of the interventions, nor on the experiences, for older adults. Furthermore, due to missing data and heterogeneity of the sample, the sample sizes for some of the occupational groups was small, so care should be taken in the interpretation or generalization of these results. Further research that looks specifically at staff behavior between settings or occupations with a larger sample would provide greater insight into different patterns of use.

In 2016 approximately 34% of aged care workers in Australia were migrants, and possibly from culturally and linguistically diverse (CALD) backgrounds (Mavromaras et al., 2017). In the present study, only 4% of participants reported to speak a language other than English at home. Those from CALD backgrounds may have chosen not to participate given the language requirements of the study, and therefore the sample might not be representative of the population of workers from CALD backgrounds.

Despite these limitations, this study is the first to explore the views and practices of aged care workers and volunteers in Australia in regards to VRT and MRT. These findings begin to establish how these interventions are used in “*treatment as usual*” paradigms, and will help to drive future research decisions and staff training based on existing clinical use. Given the high degree of use and lack of education in these interventions that was identified by the staff respondents, further investment in staff training programs could be beneficial for both the workforce, care organizations and older adults. The data also provides indicators for the clinical situations in which MRT may be useful as an intervention over and above VRT (e.g., as a compensatory strategy). Using a mixed-method analysis approach allowed for both richness and depth in the data, and has allowed for a detailed exploration of the extent to which VRT and MRT are used by the care workforce, the methodology of the interventions, and staff perceptions on their use.

## Conclusion

Reminiscence therapy and music-assisted reminiscence therapy are interventions that can improve the wellbeing of older people and the care practices of staff who care for them. This study described how these interventions were being used in clinical practice by care staff across occupational groups, with both VRT and MRT frequently used, particularly *simple* reminiscence (e.g., thematic discussions) that is spontaneous and integrated in daily care (e.g., during other activities). These findings can inform the development of future research protocols investigating the use of these interventions as treatment as usual, and to inform the practices of those working with older adults to improve the care standards.
